# Circular RNA–MicroRNA–MRNA interaction predictions in SARS-CoV-2 infection

**DOI:** 10.1515/jib-2020-0047

**Published:** 2021-03-17

**Authors:** Yılmaz Mehmet Demirci, Müşerref Duygu Saçar Demirci

**Affiliations:** Faculty of Engineering, Engineering Science Department, Abdullah Gül University, 38080 Kayseri, Turkey; Faculty of Life and Natural Sciences, Bioinformatics Department, Abdullah Gül University, 38080 Kayseri, Turkey

**Keywords:** circRNA, gene regulation, machine learning, miRNA, SARS-CoV-2

## Abstract

Different types of noncoding RNAs like microRNAs (miRNAs) and circular RNAs (circRNAs) have been shown to take part in various cellular processes including post-transcriptional gene regulation during infection. MiRNAs are expressed by more than 200 organisms ranging from viruses to higher eukaryotes. Since miRNAs seem to be involved in host–pathogen interactions, many studies attempted to identify whether human miRNAs could target severe acute respiratory syndrome coronavirus 2 (SARS-CoV-2) mRNAs as an antiviral defence mechanism. In this work, a machine learning based miRNA analysis workflow was developed to predict differential expression patterns of human miRNAs during SARS-CoV-2 infection. In order to obtain the graphical representation of miRNA hairpins, 36 features were defined based on the secondary structures. Moreover, potential targeting interactions between human circRNAs and miRNAs as well as human miRNAs and viral mRNAs were investigated.

## 1 Introduction

MicroRNAs (miRNAs) are noncoding RNAs involved in post-transcriptional gene regulation. The precursor miRNAs (pre-miRNAs) fold into characteristic hairpin structures that are used as the primary feature source in many bioinformatics approaches [1]. Another class of noncoding and endogenous RNAs is circular RNAs (circRNAs) that are generated by a unique splicing reaction known as back-splicing [2]. CircRNAs seem to be expressed in a widespread manner and they have important functions in regulation especially as sponges providing binding sites for miRNAs and RNA binding proteins [3] and a player in the regulation of alternative splicing [4].

According to the competitive endogenous RNA (ceRNA) hypothesis, RNA transcripts such as circRNAs, messenger RNAs (mRNAs), and long non-coding RNAs, include miRNA response elements and these are in competition among themselves for miRNA binding to be able to regulate the expression of each other [5]. Previous studies showed that not only miRNA but also circRNA expressions were changed during infections of both DNA and RNA viruses [6]. Although there is not much information about circRNAs’ roles during infection of emerging Severe acute respiratory syndrome coronavirus 2 (SARS-CoV-2), another member of coronaviruses, Middle East respiratory syndrome coronavirus (MERS-CoV) infection resulted in expression changes of host circRNAs [3].

In this study, we used available differentially expressed miRNA information of SARS-CoV-2 infected cells to build a machine learning based model for prediction. In addition, a comprehensive circRNA-miRNA-mRNA targeting network analysis is performed to identify biologically significant processes in SARS-CoV-2 infection. Our results show that various cellular processes including apoptosis might be affected by the competition of cellular and viral RNAs. These findings could increase the perceptions of infection through RNA-mediated host–virus interactions and lead to development of new strategies for antiviral agents.

## 2 Related works

Various studies attempted to identify human miRNAs that could target viruses [7], [8], [9], [10]. Although there are not many experimentally validated examples of miRNAs encoded by RNA viruses, computational predictions show that SARS-CoV-2 genome could produce miRNAs that could target human mRNAs [11].

Currently there is not much information about the differences in expression levels of miRNAs during SARS-CoV-2 infection. It has been shown that, highly pathogenic MERS-CoV infection causes substantial changes in the expression of many host cell circRNAs, miRNAs, and mRNAs [3].

## 3 Architecture/implementation/workflow

All data analysis, machine learning and prediction workflows were generated by using the Konstanz information miner (KNIME) platform [12]. MiRNA – target predictions were performed by using psRNATarget tool [13].

### 3.1 Graphical representation of RNA secondary structures

**Figure 1:**
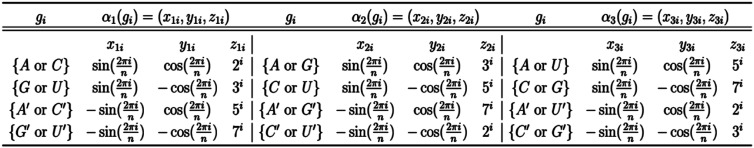
The definition of three maps.

An RNA sequence could include four bases (A, G, C, and U) that can form base pairs such as A–U, G–C, and G–U. RNAfold software from the Vienna package was used with default setting to create secondary structures [14]. For better representation, the nucleotides involved in base pairs are shown as A, G, C, and U in Figure 1, while non-base paired ones are shown as A′, G′, C′, and U′, respectively. The workflow generated in KNIME uses RNA sequence and dot-bracket representations of secondary structure to modify bases of the sequence as uppercase and lowercase characters [15].

Zhang et al. created a dynamic 3D graphical representation for RNA structure based on the chemical properties of the bases [16]

– amino group M = {A, C} and keto group K = {G, U},
– purine group R = {A, G} and pyrimidine group Y = {C, U}
– weak group H-bonds W = {A, U} and strong H-bonds group S = {C, G}.

We used the same base grouping scheme and defined three maps α1, α2 and α3 (Figure 1), where *n* is the length of the hairpin sequence and i is the index of base in the sequence.

**Figure 2:**
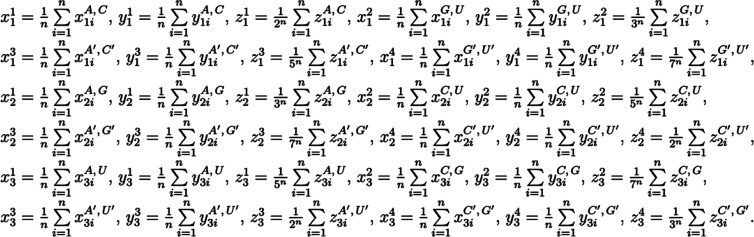
The components of 36-dimensional vector.

In order to represent miRNA hairpin secondary structure as vectors, based on the definitions from Figure 1, 36-dimensional vector was calculated as shown in Figure 2.

### 3.2 Data sets

Human miRNA sequences were obtained from MiRBase (Release 22.1) [17], human circRNA data set was downloaded from circAtlas 2.0 [18], SARS-CoV-2 CDS were based on RefSeq_NC_045512.2 from NCBI. Differentially expressed miRNA list was based on the results of Chow and Salmena [19] with some changes, since their list is composed of mature miRNAs, we used the hairpin sequences of those available (Table 1).

**Table 1:** The list of miRNAs used for training of differential expression prediction.

Regulation type	MiRNAs
Upregulated	hsa-mir-4485, hsa-mir-483, hsa-mir-6891, hsa-mir-4284, hsa-mir-4463,
	hsa-mir-155, hsa-mir-107, hsa-mir-29b-2, hsa-mir-139, hsa-mir-299,
	hsa-mir-501, hsa-mir-4745, hsa-mir-12136
Downregulated	hsa-let-7a-1, hsa-let-7a-2, hsa-let-7a-3, hsa-mir-374a, hsa-mir-194-1,hsa-mir-194-2,
	hsa-mir-4454, hsa-mir-135b, hsa-mir-16-2, hsa-mir-23b, hsa-mir-21, hsa-let-7f-1,
	hsa-mir-429, hsa-mir-5701-1, hsa-mir-5701-2, hsa-mir-5701-3, hsa-mir-450b,
	hsa-mir-7-1, hsa-mir-26b, hsa-mir-23c, hsa-mir-374c, hsa-mir-374b,
	hsa-mir-26a-1, hsa-mir-365a, hsa-mir-365b, hsa-mir-940, hsa-mir-362,
	hsa-mir-1275, hsa-mir-1296, hsa-mir-126, hsa-mir-548d-2

**Figure 3:**
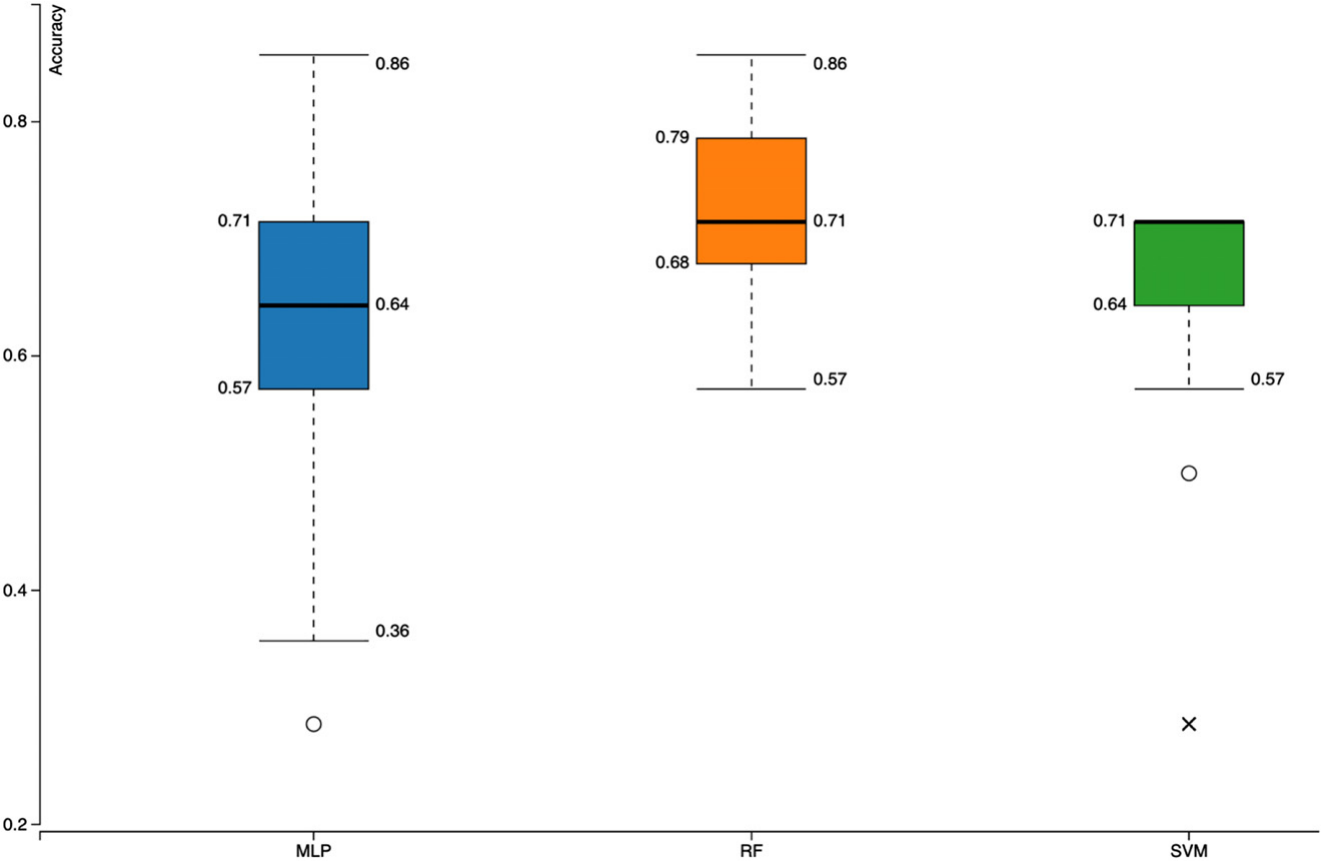
Box-plots of classi-fiers’ accuracy values.

## 4 Results

The differential expression prediction workflow was created by using 70% learning and 30% testing ratios and three different classifiers; random forest (RF), support vector machine (SVM) and multilayer perceptron (MLP) were trained with 100-fold MCCV [20] (Figure 3).

Among 2654 mature human miRNAs available in miRBase, 2498 were involved in 272,822 total targeting events with 18,950 human genes; 2498 were involved in 393,877 total targeting events with 208,642 circRNAs and 484 miRNAs targeted 11 SARS-CoV-2 genes. Some of the miRNAs reported as differentially expressed in Calu3 cells infected with SARS-CoV-2 or mock from GSE148729 did not have any predicted targets (Table 2).

**Table 2:** Number of targets of differentially expressed miRNAs on human genes (Gene), human circRNAs (CircRNA) and SARS-CoV-2 coding sequences.

MiRNA	Gene	CircRNA	SARS-CoV-2	Regulation
hsa-miR-6891-5p	197	200	1 (ORF3a)	Up
hsa-miR-4284	–	–	–	Up
hsa-miR-4463	–	–	–	Up
hsa-miR-12136	–	–	–	Up
hsa-miR-181-5p	–	–	–	Up
hsa-miR-126-5p	130	193	1 (ORF1ab)	Down
hsa-miR-194-5p	76	132	1 (ORF1ab)	Down
hsa-miR-374a-3p	100	155	2 (ORF1ab, S)	Down
hsa-miR-181-3p	–	–	–	Down
hsa-miR-1275	–	–	–	Down

**Figure 4:**
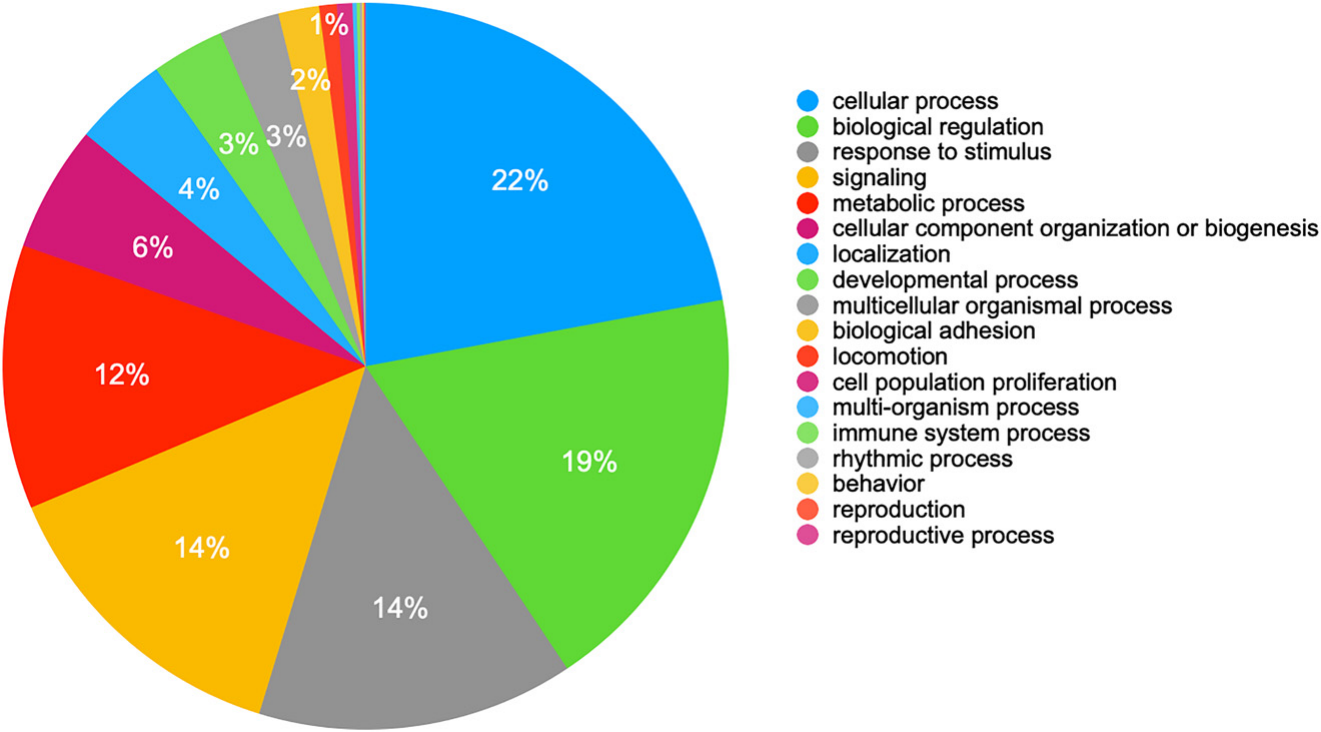
Pie-chart for the biological processes of human genes that could be targeted by hsa-miR-6891-5p. Labels on the right part are sorted in descending order making the chart direction in a clockwise order.

Upregulated human miRNA hsa-miR-6891-5p might target not only human genes and circRNAs but also ORF3a gene of SARS-CoV-2 (Table 2). PANTHER Gene Ontology analysis [21] of human gene targets showed that various biological processes could potentially be affected by the actions of this miRNA (Figure 4).

## 5 Discussion

Inter-kingdom communication mechanisms mediated by RNAs have been investigated for several organisms including a variety of viruses, *Toxoplasma gondii* (protozoan eukaryotic parasite) [22], *Histoplasma capsulatum* (infectious fungus) [23]. Viruses are parasites that depend on their host for many of their processes. Usually viral infections result in alterations of cellular pathways to modulate viral gene expression and/or accommodate virus in a favourable environment. In some cases, e.g. SARS-CoV-2 infection, host post-transcriptional gene regulation elements like miRNAs might also show differential expression levels during infection [19]. In this study, we analysed such human miRNAs (Table 1) to build a machine learning based workflow that might be used for prediction of expression changes of miRNAs during SARS-CoV-2 infection. Among the 300 models generated, the highest accuracy value was observed with RF classifier (Figure 3). While applying machine learning approaches to miRNA datasets, there are various elements that would affect the overall performance [24]. Among them, feature sets [25], [26] and the quality of data [27] might be the most important parts. When there are more datasets available, the workflow can be easily updated to include them and it is also possible to use this workflow for any kind of differentially expressed miRNAs.

There is not much known about the individual functions of circRNAs but they are acknowledged as sponges providing binding sites for miRNAs and some RNA-binding proteins [28]. The activities of host circRNAs have been investigated in Hepatitis C virus-infected cells [6] and MERS-CoV infection [3]. We performed a comprehensive target prediction analysis for human miRNAs to measure their capacity to bind human mRNAs, human circRNAs and SARS-CoV-2 genes. Based on the results represented in Table 2, SARS-CoV-2 ORF3a is the only viral target for upregulated human miRNAs. Since ORF3a protein is associated with apoptosis which is an essential mechanism for host antiviral defence to control viral infection [29], upregulation of hsa-miR-6891-5p might be crucial to decrease ORF3a expression during certain stages of infection.

Out of 2498 miRNAs that have predicted targets, 2448 had more targets in circRNAs, 27 had more in mRNAs and 23 miRNAs had equal number of targets in both groups. If the mRNA and circRNA targets of specific miRNAs are coexpressed there might be a competition for miRNA binding and considering the wide range of biological processes of a single miRNA’s targets (Figure 4) circRNA-miRNA-mRNA network could play important roles in overall gene expression especially when there is a new set of genes as target candidates during viral infections.
